# Maternal Hypotension during Fetoscopic Surgery: Incidence and Its Impact on Fetal Survival Outcomes

**DOI:** 10.1155/2013/709059

**Published:** 2013-10-21

**Authors:** Pornswan Ngamprasertwong, Mounira Habli, Anne Boat, Foong Yen Lim, Hope Esslinger, Lili Ding, Senthilkumar Sadhasivam

**Affiliations:** ^1^Department of Anesthesia, Cincinnati Children's Hospital Medical Center, 3333 Burnet Avenue, Cincinnati, OH 45229, USA; ^2^The Fetal Care Center, Cincinnati Children's Hospital Medical Center, 3333 Burnet Avenue, Cincinnati, OH 45229, USA; ^3^Division of Maternal-Fetal Medicine, Department of Obstetric and Gynecology, Good Samaritan Hospital, 375 Dixmyth Avenue, Cincinnati, OH 45220, USA; ^4^Division of Pediatric General and Thoracic Surgery, The Fetal Care Center, Cincinnati Children's Hospital Medical Center, 3333 Burnet Avenue, Cincinnati, OH 45229, USA; ^5^Division of Biostatistics and Epidemiology, Cincinnati Children's Hospital Medical Center, 3333 Burnet Avenue, Cincinnati, OH 45229, USA

## Abstract

In this retrospective cohort study, we aimed to determine the incidence of intraoperative maternal hypotension during fetoscopic surgery for twin-twin transfusion syndrome (TTTS) and to evaluate the impact of intraoperative hypotension on fetal survival. A total of 328 TTTS patients with recipient twin cardiomyopathy who underwent fetoscopic surgery under epidural anesthesia were included. The exposure of interest was maternal medical therapy with nifedipine for the treatment of fetal cardiomyopathy. We found that intraoperative hypotension occurred in 53.4% (175/328 patients). There was no statistically significant difference in incidence of hypotension between nifedipine exposure and nonexposure groups (54.8% versus 50.8%, *P* = 0.479). However, the nifedipine exposure group received a statistically significant higher dose of phenylephrine (7.04 ± 6.38 mcg/kg versus 4.70 ± 4.14 mcg/kg, *P* = 0.018) and higher doses of other vasopressor, as counted by number of treatments (6.06 ± 4.58 versus 4.96 ± 3.42, *P* = 0.022). There were no statistically significant differences in acute fetal survival rate (within 5 days) and fetal survival rate at birth between hypotensive and nonhypotensive patients. We concluded that preoperative exposure to nifedipine resulted in increased intraoperative maternal vasopressor requirement during fetoscopic surgery under epidural anesthesia. In patients who had intraoperative maternal hypotension, there was no correlation between the presence of maternal hypotension and postoperative fetal survival.

## 1. Introduction

Twin-twin transfusion syndrome (TTTS) is a serious complication of monozygotic twin pregnancy. Due to uneven sharing of placental blood flow through communicating vessels between the twins, the donor twin develops hypovolemia, oliguria, and oligohydramnios, while the recipient twin develops fluid overload, hypertensive cardiomyopathy, and heart failure [1]. TTTS is a progressive disease, and if left untreated, it will advance to severe TTTS. The fetal survival rate for advanced stage TTTS is less than 10% [2]. Selective fetoscopic laser photocoagulation (SFLP) of the aberrant placental vascular anastomoses improves the overall survival rates to 57–77% and is now considered a standard treatment for this condition [3].

Hypertensive cardiomyopathy is common in the recipient twin and is a major cause of fetal death. A recent study demonstrates that maternal medical therapy with nifedipine in combination with SFLP improves recipient twin survival from 75% to 83% [4]. This antihypertensive drug is administered to the mother for the treatment of hypertensive cardiomyopathy in the fetus. Therefore, this raises concern that preoperative nifedipine may cause hypotension in the mother who is usually normotensive, especially when intraoperative hypotension is a common complication in parturients undergoing regional anesthesia. Maternal hypotension poses multiple risks during delivery. For the mother, reported unfavorable outcomes from intraoperative hypotension include nausea, vomiting, unconsciousness, and aspiration [5]. For the baby, maternal hypotension diminishes uteroplacental blood flow and may result in neonatal hypoxia and acidosis [5]. 

Various anesthetic techniques can be utilized for SFLP, including monitor anesthetic care, sedation, regional anesthesia, and general anesthesia. Epidural anesthesia is a commonly utilized anesthetic method for SFLP performed frequently in the second trimester at our institution. However, little is known about the incidence and consequences of intraoperative maternal hypotension during SFLP and its effect on fetal survival.

The aim of our study is to determine the effects of preoperative nifedipine use in normotensive mothers on the incidence of maternal hypotension during SFLP under epidural anesthesia. We also aim to study the impact of intraoperative hypotension on fetal survival outcomes after SFLP. 

## 2. Materials and Methods

This study was reviewed and approved by the Institutional Review Board of Cincinnati Children's Hospital Medical Center (IRB #2010-1979). The requirement for written informed consent was waived by the Institutional Review Board.

### 2.1. Design and Study Population

In this retrospective cohort study, medical records, anesthetic records, and database of patients with diagnosis of TTTS who underwent SFLP at the Fetal Care Center of Cincinnati during April 2004–July 2010 were reviewed. The diagnosis of TTTS was based on a monochorionic-diamniotic twin pregnancy (single placenta, thin dividing membrane, same gender) that was complicated by polyhydramnios in the recipient (>8 cm depth of amniotic fluid) and oligohydramnios in the donor (<2 cm depth of amniotic fluid), with the exclusion of other causes for amniotic fluid and growth discrepancy. Inclusion criteria included TTTS patients with recipient twin cardiomyopathy who underwent SFLP under epidural anesthesia. Exclusion criteria included patients who received general anesthesia, monitor anesthetic care, sedation, spinal anesthesia, or combined spinal and epidural anesthesia. We excluded patients with preoperative diagnosis of maternal hypertension, triplets, or higher order multiple gestation. Patients were also excluded if the fetuses had congenital anomalies or chromosomal anomalies.

Severity of disease was classified into stages based on the degree of cardiomyopathy in the recipient twin. The stage is determined by fetal echocardiographic findings of three parameters: the presence and severity of atrioventricular valvular regurgitation, ventricular wall hypertrophy, and ventricular function as assessed by the myocardial performance index (MPI). The MPI is a Doppler index indicative of both systolic and diastolic myocardial functions. It is defined as the sum of the isovolumic relaxation and the isovolumic contraction times divided by ejection time, measured from the Doppler inflow and outflow spectral profiles. The details in classification of fetal cardiomyopathy in TTTS have previously been described [6]. The severity of recipient TTTS cardiomyopathy is scored based on the most severe findings of the three parameters above. The Cincinnati staging system, which integrates the severity of cardiomyopathy into IIIa (mild cardiomyopathy), IIIb (moderate cardiomyopathy), IIIc (severe cardiomyopathy), and IV (hydrops), was used to compare the severity of TTTS in this study. 

### 2.2. Outcome Variables

Clinical records including maternal demographics, medical history, and neonatal survival data were reviewed in all patients if available. Patients who were exposed to nifedipine received at least one dose of nifedipine 20 mg before surgery and then received 20 mg of nifedipine postoperative every 6 hours until all fetal echocardiographic findings of cardiomyopathy resolved or the patients delivered. The primary dependent variable was intraoperative maternal hypotension, as a binary outcome. Intraoperative maternal hypotension is defined as maternal systolic blood pressure 20% lower than baseline and an absolute value less than 90 mmHg [5]. Blood pressure was measured by an oscillometric device and recorded at least every five minutes. The secondary dependent variables are the severity and duration of maternal hypotension and vasopressors administration (amount and number of treatments). Other covariates include gestational age at SFLP, gestational age at birth, classification by Cincinnati staging system, maternal age, maternal body weight, amount of local anesthetic given, amount of intraoperative intravenous fluid given, surgical time, and anesthesia time. Surgical time was defined as time from skin incision to skin closure. Anesthesia time was defined as time from the first bolus of epidural block to skin closure. 

We also explored the impact of intraoperative maternal hypotension on fetal survival rates. Since a previous study [4] demonstrated a different survival rate for the recipient twin in patients who were exposed and not exposed to nifedipine, only nifedipine exposure patients were included in this part of the analysis. The acute survival rate of the recipient and donor twin was determined by ultrasound or fetal echocardiogram performed on days 3–5 after SFLP [4]. The survival rate at birth of the recipient twin and donor twins was defined as live birth at delivery.

### 2.3. Statistical Analysis

In order to reduce bias, the investigators first reviewed all the anesthetic records without knowledge of maternal exposure to preoperative nifedipine and fetal survival. Study data were collected and managed using REDCap (Research Electronic Data Capture) electronic data capture tools hosted at Cincinnati Children's Hospital Medical Center [7]. REDCap is a secure, web-based application designed to support data capture for research studies. After the intraoperative data was collected and inputted into the REDCap database, medical records and Fetal Care Center database information were reviewed and filled into a separate data record form. The intraoperative REDCap database and the data from medical records/Fetal Care Center were then matched using study identification numbers. 

Even though the general incidence of intraoperative hypotension during epidural anesthesia for SFLP is unknown, we assumed that the incidence is about 50% as reported in cesarean section cases [5]. In order to detect 20% difference in the incidence of hypotension in patients undergoing epidural anesthesia for SFLP who were exposed and unexposed to preoperative nifedipine with an *α* of 0.05 and 80% power, a sample size of 121 subjects in each group was estimated.

Statistical analysis was performed using SAS software, (Version 9.2, Cary, NC). Hypotensive events in patients who were exposed to nifedipine compared to those who were not exposed were analyzed using the Chi square test. For secondary endpoints, statistical analysis was performed with the Chi square, Fisher's exact, or Cochran-Mantel-Haenszel (CMH) tests for categorical data and with the *t*-test or analysis of variance (ANOVA) test for continuous variables. The fetal survival outcomes between hypotensive and normotensive patients were compared using the Chi square and CMH tests. Statistical significance was considered to be present if  *P*  values ≤0.05.

## 3. Results

### 3.1. Baseline Characteristic of Study Population

**Figure 1:**
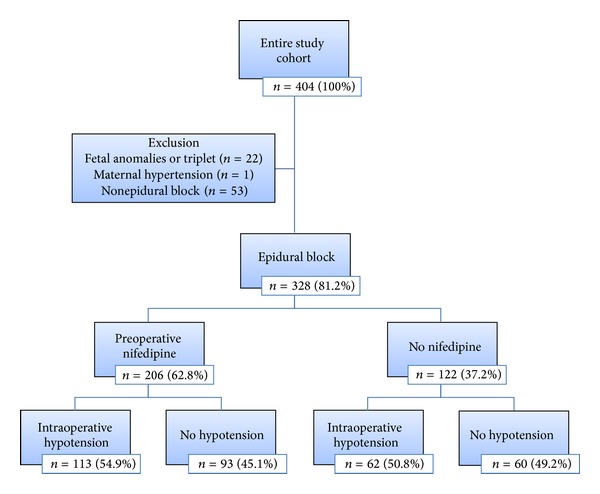
Flow chart illustrates subject enrollment and analysis. A total of 328 subjects were included for analysis in this retrospective cohort study. There was no difference in the incidence of intraoperative maternal hypotension with preoperative nifedipine in comparison with the without preoperative nifedipine (54.8% versus 50.8%, *P* = 0.479).

There were 404 patients undergoing SFLP during April 2004–July 2010. From the entire cohort, 328 subjects were included into this study (Figure 1). Among 328 patients, preoperative nifedipine was administered in 206 patients (62.8%), and the remaining 122 patients (37.2%) did not receive preoperative nifedipine. Intraoperative hypotension occurred in 53.4% of the patients undergoing SFLP under epidural anesthesia (175/328 patients). There were no clinically and statistically significant differences in maternal and fetal demographic characteristics between patients who developed and did not develop intraoperative hypotension as demonstrated in Table 1. There was no significant difference in volume of local anesthetic agent administered, and intravenous fluid given during intraoperative period was limited to less than 250 mL during the study period.

### 3.2. Effect of Preoperative Nifedipine on Intraoperative Outcomes

**Figure 2:**
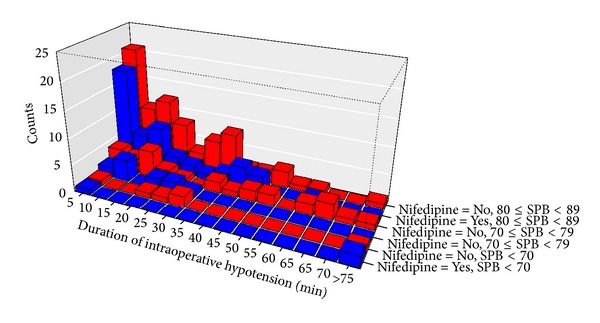
Severity and duration of intraoperative maternal hypotension in the patients during selective fetoscopic laser photocoagulation under epidural anesthesia. *y*-axis represented the number of patients in nifedipine exposure group (blue color) and nonexposure to nifedipine group (red color). *x*-axis represented the duration of hypotension in minutes.

There was no statistically significant difference in incidence of hypotension between nifedipine exposure and nonexposure groups (54.8% versus 50.8%, *P* = 0.479). There was no statistical difference in duration and severity of intraoperative hypotension between nifedipine exposure and nonexposure groups as demonstrated in Table 2 and Figure 2. A minority of patients in the nifedipine (*n* = 3) and nonexposure to nifedipine (*n* = 1) groups developed severe hypotension for a prolong period of time (Figure 2).

 Vasopressors including ephedrine and phenylephrine were given to maintain maternal blood pressure in almost all patients (200/206, 97.1% in nifedipine exposure group and 119/122, 97.5% in nonexposure to nifedipine group). These vasopressors were administered when maternal blood pressure dropped from baseline, although administration of vasopressors occurred even before the study criteria for maternal hypotension were met. Despite aggressive treatment with vasopressors, hypotension still occurred in 54.8% and 50.8% (*P* = 0.479) of nifedipine exposure and nonexposure groups, respectively.

Ephedrine was more commonly administered than phenylephrine for treatment of maternal hypotension in our study patients. There was no statistically significant difference in the ephedrine dose given to the nifedipine exposure group versus nonexposure group. However, the nifedipine exposure group received a statistically significant higher dose of phenylephrine (7.04 ± 6.38 mcg/kg versus 4.70 ± 4.14 mcg/kg, *P* = 0.018) and higher doses of total vasopressors, as counted by a number of vasopressor treatments (6.06 ± 4.58 versus 4.96 ± 3.42, *P* = 0.022). 

### 3.3. Effect of Intraoperative Hypotension on Fetal Survival Outcomes

In order to minimize the potential confounding effect of nifedipine on fetal survival rate, only patients exposed to preoperative nifedipine (*n* = 206) were included in this analysis. There were no statistically significant differences in maternal and fetal demographic data between hypotensive and Nonhypotensive patients (Table 3). Vasopressors were administered in 94.6% of Nonhypotensive patients to maintain blood pressure as the maternal blood pressure decreased in this group, but with prompt treatments, the study criteria for hypotension were not met. Nonhypotensive patients, however, required lower doses of vasopressors, as counted by number of treatments when compared with hypotensive patients (3.42 ± 2.37 versus 8.24 ± 4.82, *P* < 0.001). We found no statistically significant differences in acute fetal survival rate and fetal survival rates at birth between patients who developed and did not develop intraoperative maternal hypotension (Table 4). 

## 4. Comment

This study demonstrated that maternal preoperative exposure to nifedipine resulted in a higher intraoperative vasopressor requirement to maintain maternal blood pressure during SFLP under epidural anesthesia. With aggressive vasopressor treatment of maternal blood pressure, we found no difference in incidence of intraoperative maternal hypotension between patients who were exposed and not exposed to nifedipine. Aggressive and timely vasopressor treatments of intraoperative maternal blood pressure during SFLP contributed to comparable acute fetal survival and survival at birth between those who had and had not maternal hypotension intraoperatively. 

We found that intraoperative maternal hypotension is common after epidural anesthesia in second trimester parturients undergoing SFLP. In our study, the incidence of intraoperative maternal hypotension after epidural block in the second trimester is 53.8%. This is comparable to the incidence of maternal hypotension during regional block in term parturients (50–100%) [5]. Even though intravenous fluid administration is an effective method for prevention and treatment of intraoperative maternal hypotension during cesarean section [5], fluid administration during fetoscopic surgery has traditionally been limited due to the risk of maternal pulmonary edema [8]. None of the patients in our study developed pulmonary edema; nevertheless, intraoperative fluid given to these patients during the study period was limited to less than 250 mL to minimize the pulmonary edema risk in the setting of nifedipine tocolysis. Intraoperative maternal hypotension in our study was mainly treated by administration of vasopressors. 

Ephedrine and phenylephrine are both commonly used vasopressors in parturient. The superiority of one drug over another has been debated [9–11]. Ephedrine has long been used for treatment of intraoperative hypotension in parturients since it has minimal effect on uteroplacental blood flow. Phenylephrine has become popular in the recent years since it produces less fetal acidosis in patients undergoing cesarean section in comparison to ephedrine [10]. In our study, ephedrine was more frequently used than phenylephrine; however, administration of both drugs in the same patient was common. 

Nifedipine given via the mother has been used for the treatment of fetal hypertensive cardiomyopathy, in order to improve recipient twin survival rate after SFLP [4]. Nifedipine is also a tocolytic drug with 77% placental transfer [12]. It is the preferred first line tocolytic agent for treatment of preterm labor due to its effectiveness and fewer maternal side effects [13]. Nifedipine is typically given at 20 mg every 6 hour, started the night before or the morning of SFLP surgery. This raises concern for the effect of nifedipine and epidural anesthesia on maternal blood pressure in the normotensive parturient when maternal hypotension is well-known as a common complication after epidural block. Decreases in maternal blood pressure have been related to fetal hypoxia and acidosis [14]. Many studies demonstrated that hypotension secondary to nifedipine given for preterm labor in normotensive patients were minimal [15–17], but severe hypotension resulting in fetal death from nifedipine has been reported [18]. In this study, we found no significant difference in incidence or duration of maternal hypotension between patients who were exposed and not exposed to nifedipine. However, patients who were exposed to nifedipine received a higher number of vasopressor treatments. It is possible that (1) hypotension in patients exposed to nifedipine is more difficult to treat, and a higher dose of vasopressor is required or (2) hypotension is more common in nifedipine exposed group, but these patients received immediate and aggressive vasopressor treatment with restoration of normotension so that these brief hypotensive events were not be recorded with intermittent (every 3 to 5 minutes) blood pressure measurements noted in the anesthetic record.

This is the first study to examine the impact of maternal hypotension on the fetuses undergoing SFLP. The relationship of intraoperative maternal hypotension and fetal hypoxia/acidosis has been demonstrated in the parturient undergoing cesarean section [14]. Unlike a healthy full-term fetus undergoing cesarean section, the second-trimester fetuses undergoing SFLP are usually sick with cardiovascular compromise, related to anemia in the donor twin and cardiomyopathy in the recipient twin. In this study, we could not demonstrate the negative impact of intraoperative hypotension on fetal survival rate, including acute survival (within 5 days after surgery) and survival at birth. However, these survival outcomes were measured many hours after the transient intraoperative maternal hypotension occurred. During this gap, there may be non-life-threatening acute fetal deterioration; or in the meantime other confounders may occur and have effect on fetal survival rate. To eliminate this limitation, a prospective intraoperative fetal hemodynamic measurement study is required for detection of acute fetal deterioration related to maternal hypotension. 

We conclude that preoperative exposure to maternal nifedipine resulted in a higher intraoperative vasopressor requirement to maintain maternal blood pressure during fetoscopic surgery under epidural anesthesia. With aggressive and timely management with vasopressors, there was no negative impact of intraoperative maternal hypotension during SFLP on postoperative fetal survival outcomes.

## Figures and Tables

**Table 1:** Study population characteristics of nifedipine exposure group and nonexposure group. There was no statistically significant difference in maternal and fetal demographic data between these two groups.

	Nifedipine group (*n* = 206)	No nifedipine group (*n* = 122)
Maternal		
Age (yr)	29.4 ± 5.9	28.6 ± 5.5
Weight (kg)	78.7 ± 17.6	78.9 ± 19.3
Height (cm)	165.6 ± 7.5	164.5 ± 7.3
Gestational age at procedure (week)	21.2 ± 2.5	21.1 ± 2.5
Gravida	2 (1–3)	2 (2-3)
Para	1 (0–2)	1 (1-2)
Surgery time (min)	64.0 ± 23.7	76.4 ± 31.6
Anesthesia time (min)	98.5 ± 25.6	119.2 ± 36.2
Volume of local anesthetic agent given via epidural block (mL)	20.3 ± 6.3	22.6 ± 6.0
Intraoperative intravenous fluid (mL/kg)	1.68 ± 1.02	1.76 ± 1.56
Donor twin		
Gestational age at birth (week)	30.6 ± 3.8	29.9 ± 5.0
Body weight at birth (g)	1391.7 ± 614.4	1347.1 ± 659.5
Recipient twin		
Gestational age at birth (week)	30.6 ± 3.8	29.9 ± 5.0
Body weight at birth (g)	1625.4 ± 621.8	1687.4 ± 674.4

Data shown in mean ± SD, median (range), or number (%).

**Table 2:** Effect of preoperative nifedipine exposure on intraoperative outcomes.

Intraoperative events	Nifedipine group (*n* = 206)	No nifedipine group (*n* = 122)	*P* value
Intraoperative hypotension	113 (54.8%)	62 (50.8%)	0.479
Duration of intraoperative hypotension (min)	24.8 ± 21.2	19.8 ± 21.1	0.135
Number of patients received ephedrine	188 (91.3%)	113 (92.6%)	0.665
Intraoperative ephedrine (mg/kg)	0.35 ± 0.23	0.36 ± 0.24	0.822
Number of patients received phenylephrine	127 (61.6%)	61 (50.8%)	0.057
Intraoperative phenylephrine (mcg/kg)	7.04 ± 6.38	4.70 ± 4.14	**0.018**
Number of vasopressor treatment (times)	6.06 ± 4.58	4.96 ± 3.42	**0.022**

Data shown in *n* (%) or mean ± SD.

**Table 3:** Perioperative characteristics of patients with and without intraoperative hypotension. Intraoperative hypotension is defined as maternal systolic blood pressure below 80% of baseline and an absolute value less than 90 mmHg. Only nifedipine exposure patients were included to minimize confounder. The Cincinnati staging system, which integrates the severity of cardiomyopathy into IIIa (mild cardiomyopathy), IIIb (moderate cardiomyopathy), IIIc (severe cardiomyopathy), and IV (hydrops), was used to compare the severity of TTTS in this study.

	Hypotension (*n* = 113)	No hypotension (*n* = 93)	*P* value
Preoperative			
Gestational age at procedure (week)	21.4 ± 2.4	20.9 ± 2.6	0.148
Cincinnati staging			
(i) IIIa	14 (12.4%)	9 (9.7%)	0.653
(ii) IIIb	23 (20.4%)	19 (20.4%)	
(iii) IIIc	67 (59.3%)	58 (62.4%)	
(iv) IV	9 (8.0%)	7 (7.5%)	
Intraoperative			
Surgery time (min)	64.8 ± 24.3	63.0 ± 23.0	0.590
Anesthesia time (min)	100.6 ± 26.3	96.0 ± 24.6	0.206
Duration of intraoperative hypotension (min)	25.6 ± 22.9	0	
Number of patients who received intraoperative vasopressor	113 (100%)	88 (94.6%)	**0.013**
Number of vasopressor treatments	8.24 ± 4.82	3.42 ± 2.37	**<0.001**
Postoperative			
Donor twin			
Gestational age at birth (week)	30.5 ± 4.1	30.7 ± 3.4	0.713
Body weight at birth (g)	1371.1 ± 626.2	1416.6 ± 604.4	0.683
Recipient twin			
Gestational age at birth (week)	30.5 ± 4.1	30.7 ± 3.4	0.713
Body weight at birth (g)	1623.8 ± 626.8	1627.4 ± 620.4	0.973

Data shown in mean ± SD, or number (%).

**Table 4:** Fetal survival outcomes of patients with and without intraoperative hypotension. The acute survival rate of the recipient and donor twins was determined by ultrasound or fetal echocardiogram performed on days 3–5 after fetoscopic surgery. The survival rate at birth of the recipient twin and donor twins was defined as live birth at delivery.

	Hypotension (*n* = 113)	No hypotension (*n* = 92)	*P*-value
Acute fetal survival			
Donor	98 (86.7%)	84 (90.3%)	0.423
Recipient	105 (92.9%)	86 (92.5%)	0.902
At least one twin	108 (95.6%)	89 (95.7%)	0.965
Survival at birth			
Donor	85 (75.2%)	73 (78.5%)	0.580
Recipient	100 (88.5%)	86 (92.5%)	0.337
At least one twin	105 (92.9%)	89 (95.7%)	0.397

Data shown in number (%).
